# Supplementary material for: Photo-initiated solvent-mediated depolymerization of consumer poly(methyl methacrylate) without chlorinated reagents
Source: Nat Commun. 2026 Jan 28;17:1235. doi: 10.1038/s41467-025-67997-7 (PMC12864808; doi:10.1038/s41467-025-67997-7)
Supplement: Supplementary file 1 — Supplementary Information [file 41467_2025_67997_MOESM1_ESM.pdf]

Supplementary Information

Photo-initiated solvent-mediated depolymerization of consumer poly(methyl methacrylate) without chlorinated reagents

Authors: Jonathan T. Husband,*[1,2] Gavin Irvine, [1,2] Callum R. Morris,[3] Andrea Folli,[3,4] Matthew G. Davidson,*[1,2] Simon J. Freakley*[1,2]

Affiliations:

[1] Bath Institute of Sustainability and Climate Change, East Building, Claverton Down, Bath, BA2 7AY.

[2] Department of Chemistry, University of Bath, Claverton Down, Bath BA2 7AY, U.K.

[3] Cardiff Catalysis Institute, School of Chemistry, Cardiff University, Translational Research Hub, Maindy Road, Cardiff, CF24 4HQ, United Kingdom

[4] Net Zero Innovation Institute, Cardiff University, Translational Research Hub, Maindy Road, Cardiff, CF24 4HQ, United Kingdom

*Corresponding authors. Jonathan T. Husband: jth67@bath.ac.uk; Matthew G. Davidson: M.G.Davidson@bath.ac.uk; Simon J. Freakley: sf756@bath.ac.uk.

Depolymerizations

PMMA depolymerization general set-up

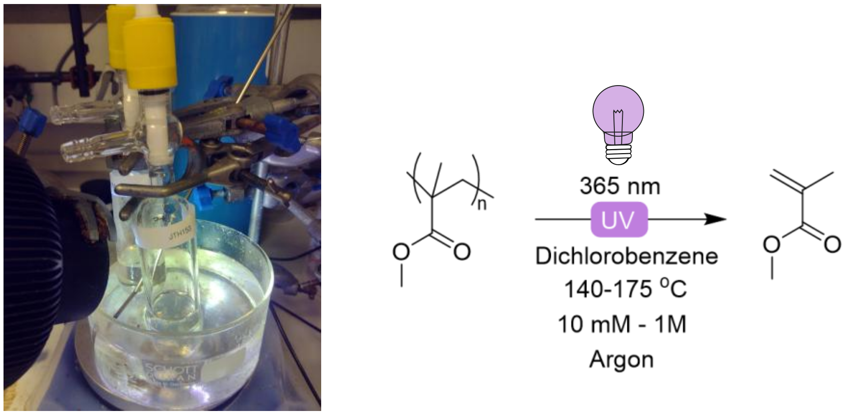

Figure S1: PMMA depolymerization set-up. Photograph of the general depolymerization set-up (left) and general scheme (right).

Lamp power

To study the effect of lamp distance on conversion the depolymerizations was undertaken at 140 ^{o}C, with the lamp at 2 cm and 6 cm. To estimate lamp output the emission of the lamp was measured by UV-A meter at various lamp distances (Figure S2). The detector had a limit of 40 mW/cm^2, so the relationship was extrapolated to distances < 10 cm. At a lamp distance of 2 cm (≈ 100 mW/cm^2), conversion was measured at 16 %, whereas at a lamp distance of 6 cm (≈ 60 mWcm^2), conversion was measured at 12 %.

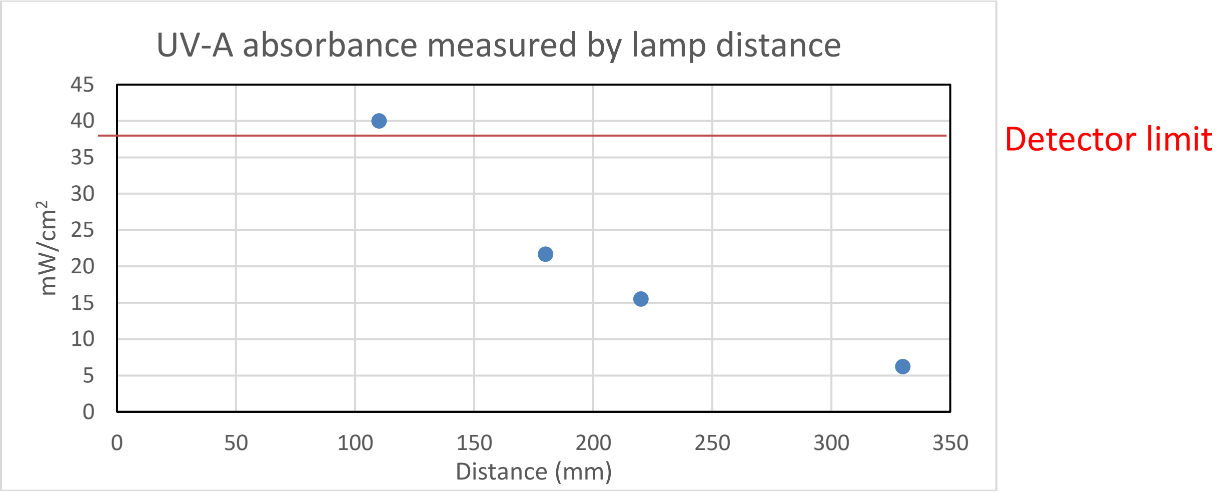

Figure S2: Study on lamp power. UV-A absorbance measured at distances from the UV lamp.

Analysis of depolymerization conversion

For monitoring, samples were run with approximately 50:50 NMR solvent to crude reaction solution. Acetone-d_6 was found to help resolve the PMMA methyl from monomer methyl. For low conversion samples the vinyl protons on the monomer could be integrated against total OMe integration/3: $Equation\ (3){:}\ Conv(\%) = \int H_{Vinyl} / (\int H_{OMe}/3) * 100$. For samples that reach higher conversion, the OMe for Monomer and OMe for Polymer can be differentiated and the following calculation can also be used: : $Equation\ (4){:}\ Conv(\%) = \int H_{OMe\ monomer} / (\int H_{OMe\ polymer}) * 100$. Both methods gave relative errors within ± 5% even with a slight overlap of peaks, therefore all reported values are with equation (1). In addition, polymer OMe protons (~3.4 ppm) can be compared to polymer CH_2-CH_3 regions at 0.7 – 1.2 ppm, and all samples maintained the ratio 1:1 indicating the integrity of PMMA architecture.

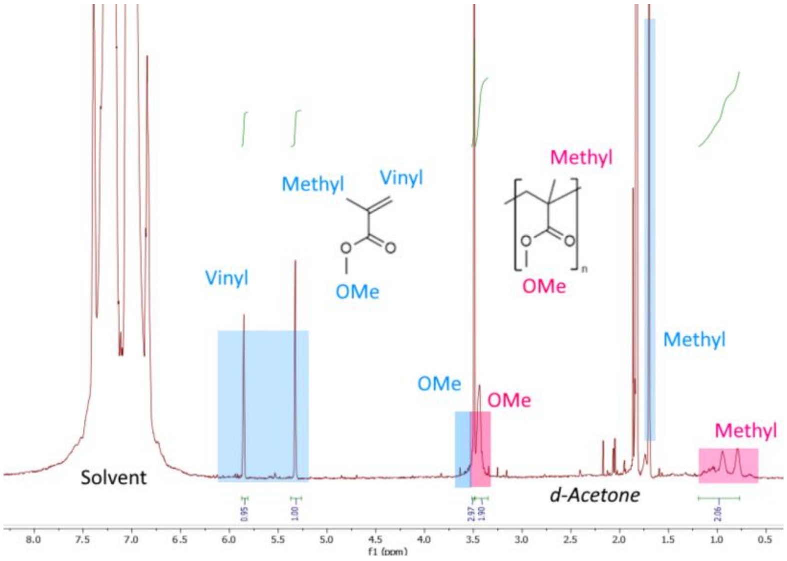

$$Equation\ (3){:}\ \frac{0.975}{4.87/3} = 60\%$$

$$Equation\ (4){:}\ \frac{2.97}{4.87} = 61\%$$

Figure S3: Example ^{1}H NMR interpretation. ^{1}H NMR (d_6-acetone) regions used for integration in a reaction that reached high conversion (blue attributed to monomer, pink attributed to polymer), showing good agreement between equations (3) and (4) for integration.

Distillation of rMMA

To recycle MMA, the crude reaction solution in dichlorobenzene was heated to around 180°C in a short path distillation set-up (Figure S4) for about 4 hours. MMA was collected in a round bottom flask, 84% of MMA was recovered from the crude NMR conversion; while some monomer remained in the distillation set-up. The distilled MMA solution (2.7 mL) was 73 mol% MMA (1.84 g) with the remainder being co-evaporated dichlorobenzene (0.96 g).

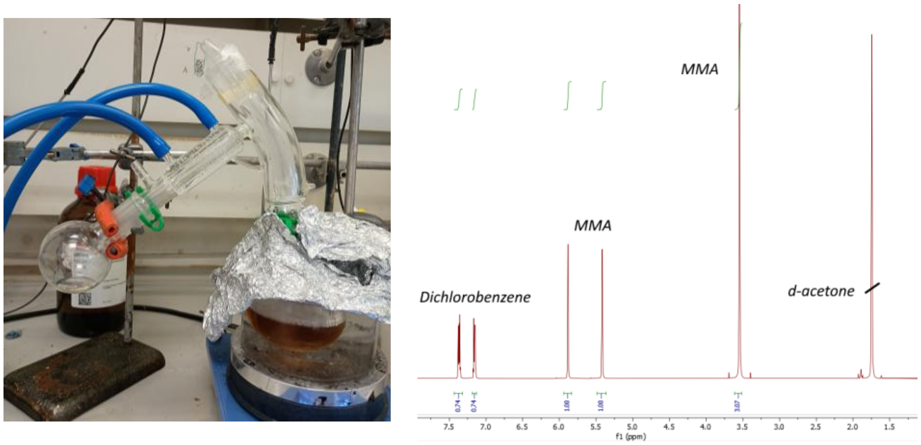

Figure S4: **Distillation of rMMA** Left: Photo of rMMA distillation set-up. Right: ^{1}H NMR (*d*$_6$-acetone) spectra of rMMA distillate.

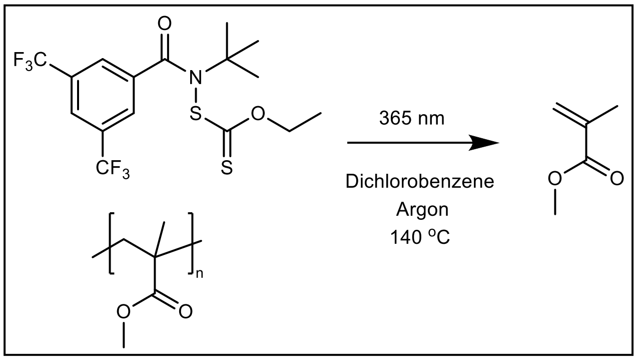

Figure S5: Reaction scheme for PMMA depolymerization with xanthylation catalyst.

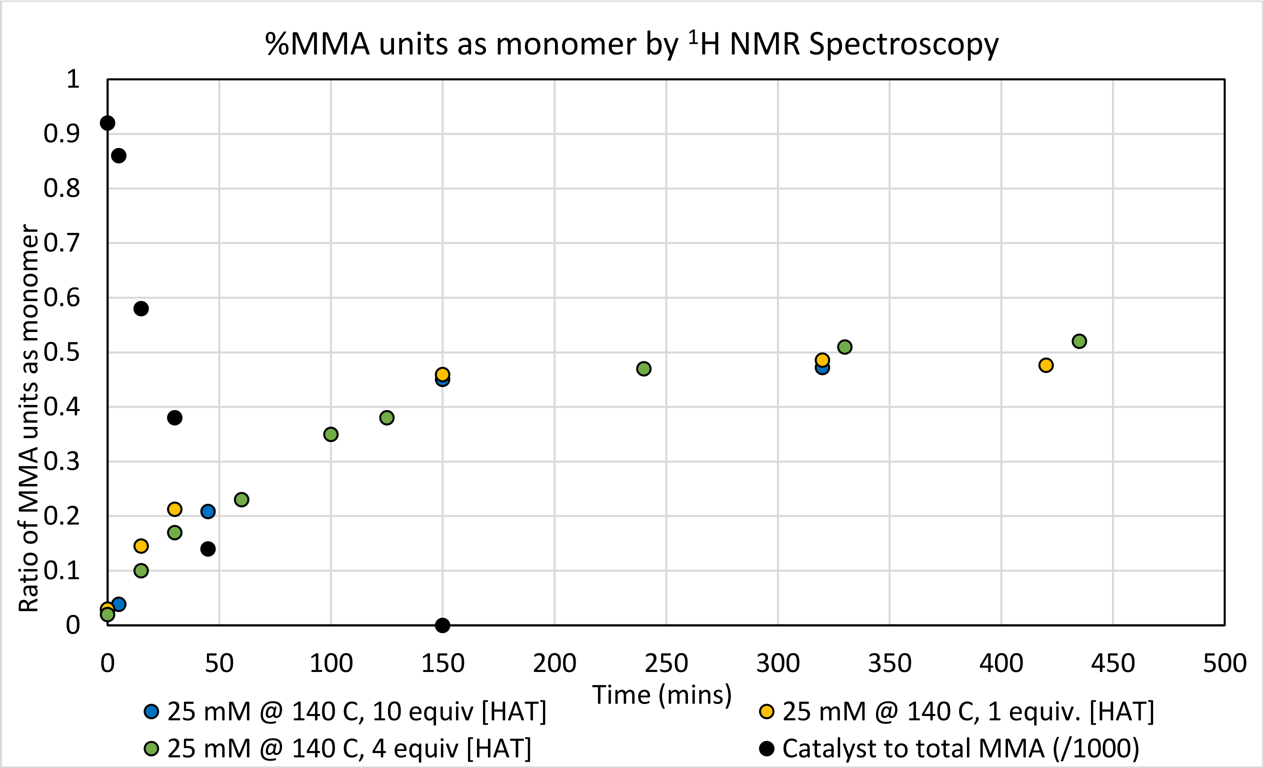

Figure S6: Conversions for PMMA depolymerization with xanthylation catalyst versus catalyst equivalents. Conversion of PMMA to monomer measured by ^{1}H NMR spectroscopy varying xanthylation catalyst loadings (10 equiv. blue, 4 equiv. green, 1 equiv. yellow), and catalyst to MMA ratio (black), showing no effect of xanthylation catalyst.

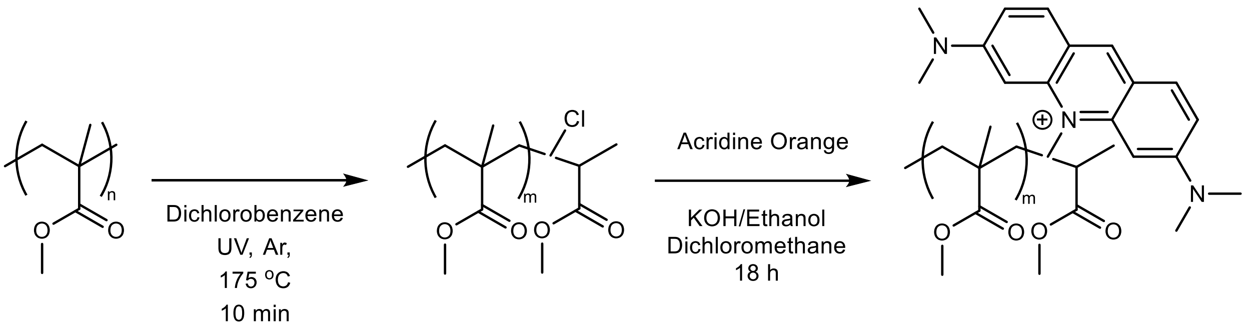

Figure S7: Reaction scheme for two-step method for acridine functionalization with initial PMMA depolymerization in dichlorobenzene.

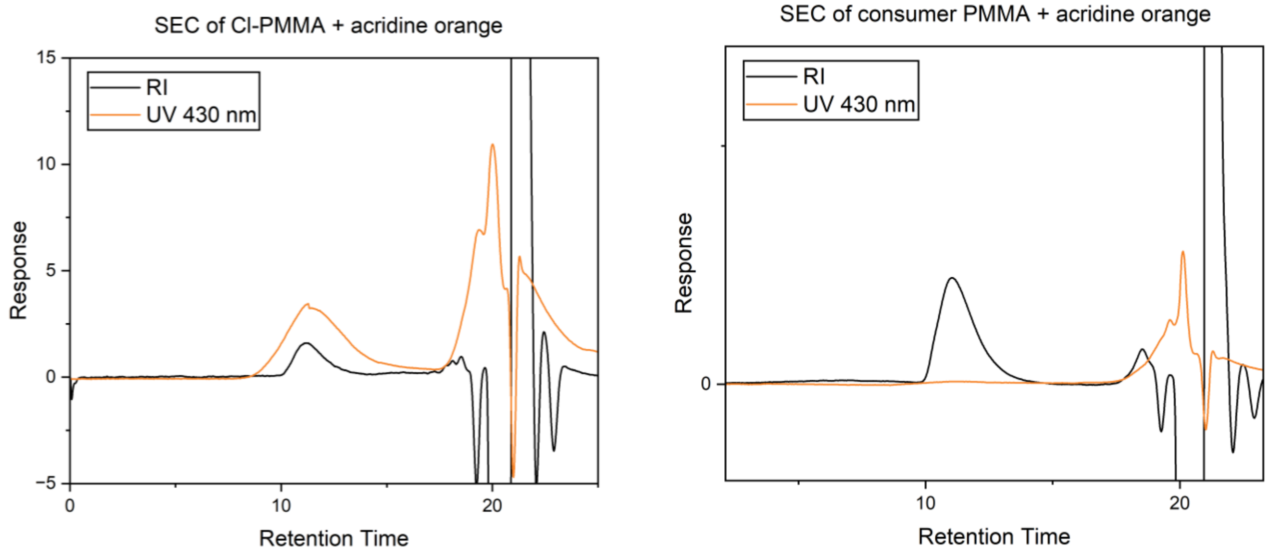

Figure S8 and 9. SEC chromatograms showing reaction of Cl-PMMA with acridine orange. SEC (THF) chromatograms for Cl-PMMA reacted with acridine orange (left) and consumer PMMA reacted with acridine orange (right), showing dramatic increase in 430 nm absorption (orange) corresponding to polymer RI response (black) in the Cl-PMMA polymer versus the consumer PMMA (right).

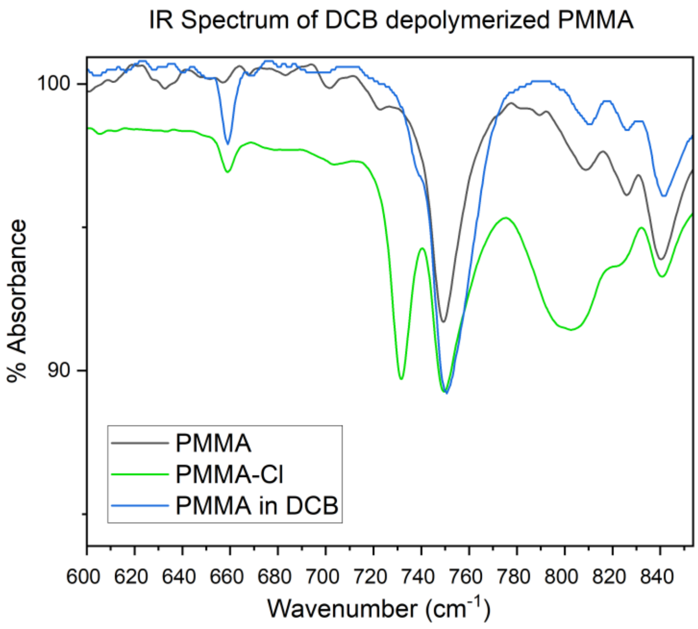

Figure S10. FT-IR showing potential PMMA-Cl absorbance. FT-IR spectrum of 200 kDa PMMA left for 1 hour under DCB depolymerization conditions (green) versus 200 kDa PMMA (black) and PMMA in DCB (blue) controls, illustrating the new potential C-Cl absorbance at 725 nm.

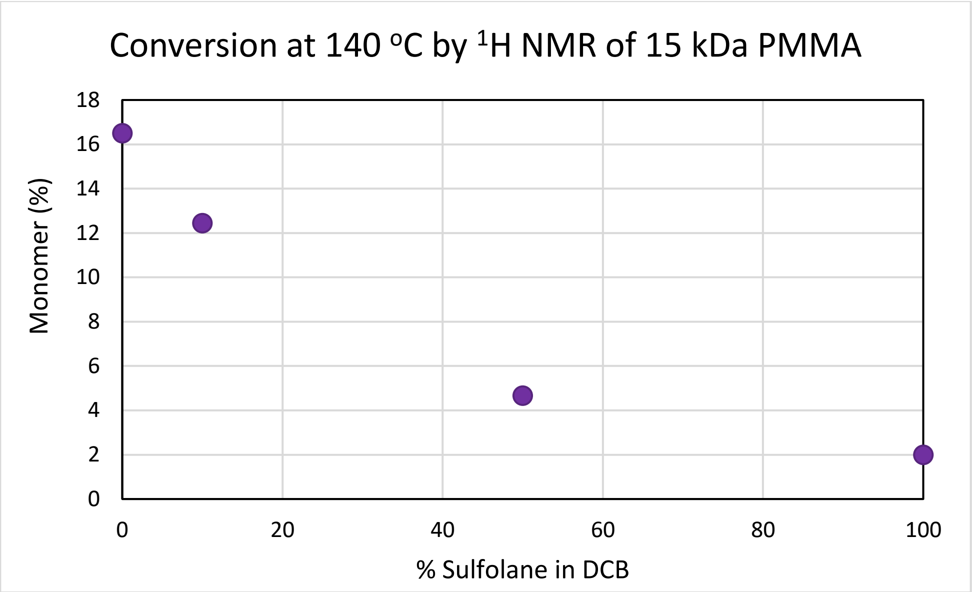

Fig S11. Depolymerization conversion versus titration of sulfolane with dichlorobenzene Measured monomer produced by ^{1}H NMR at different sulfolane DCB mixtures, showing reduction in conversion at increasing sulfolane concentrations. Reaction conducted with UV at 140 °C, 15 kDa PMMA, 4 hours. Results indicate that small additions of sulfolane to DCB decrease conversion more than a linear relationship, suggesting sulfonate-induced quenching of the depropagation is occurring.

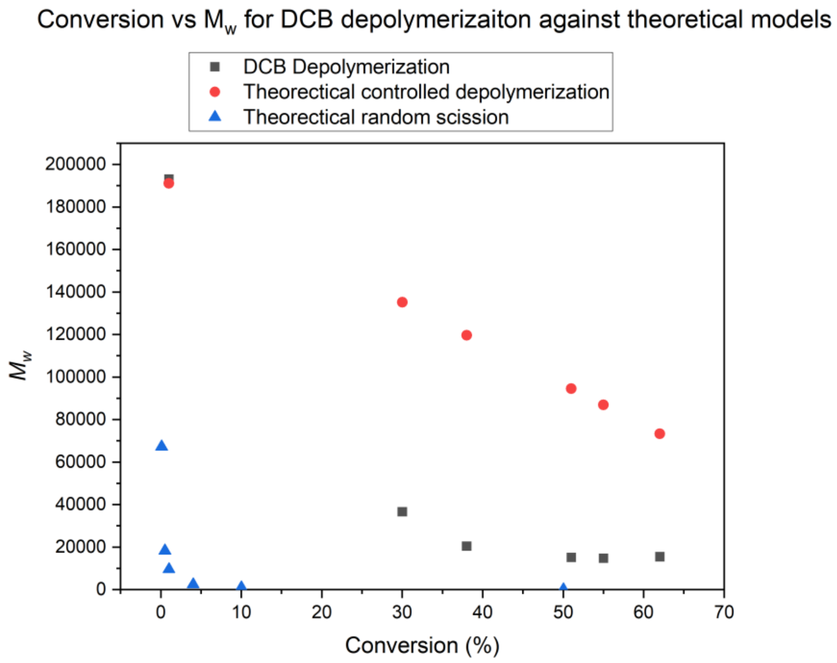

Fig S12. Conversion vs M_w for DCB PMMA depolymerization against theoretical depolymerizations. Measured monomer by ^{1}H NMR spectroscopy for depolymerizarion in DCB at 175°C, with 200 kDa PMMA (black squares), versus theoretical conversions for random scission (blue triangles) and an end chain controlled radical depolymerization (red circles).

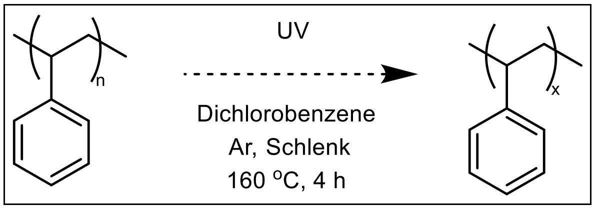

Figure S13: Reaction scheme for the attempted depolymerization of polystyrene

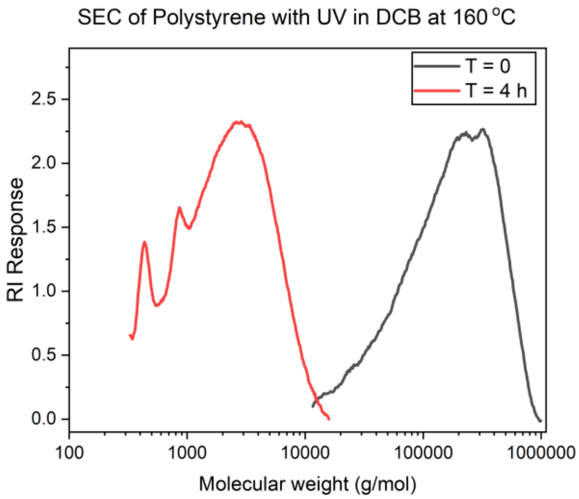

Figure S14: SEC of attempted polystyrene depolymerization. SEC (THF) chromatogram, showing reduced M_w in polystyrene subjected to depolymerization conditions (red, 160 °C, 4 hours, dichlorobenzene), versus initial polystyrene (black), calibrated to polystyrene standards.

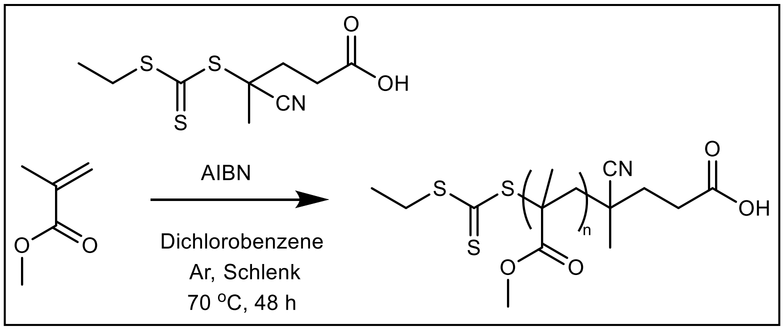

Figure S15: Reaction scheme of MMA RAFT polymerization targeting 15 kDa.

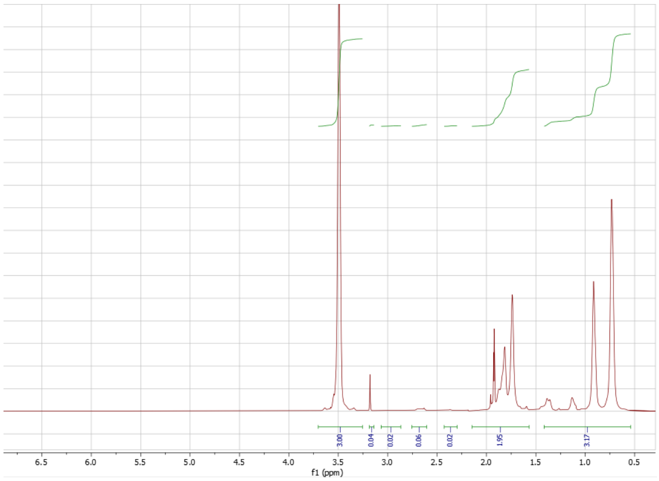

Figure S16. **^{1}H NMR of RAFT 15 kDa PMMA post-precipitation.** ^{1}H NMR of RAFT 15 kDa PMMA post-precipitation (d_6-acetone, 500 MHz)

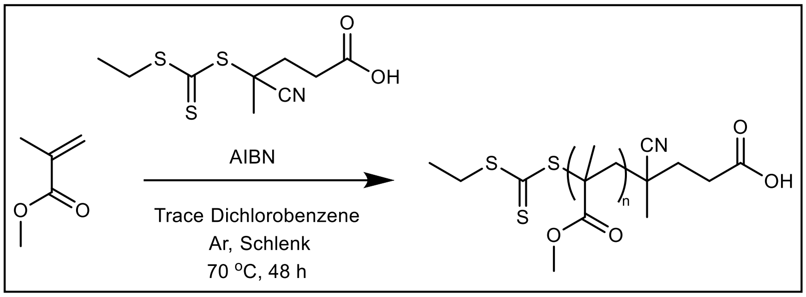

Figure S17: **Reaction scheme of recycled MMA (rMMA) RAFT polymerization targeting 15 kDa.**

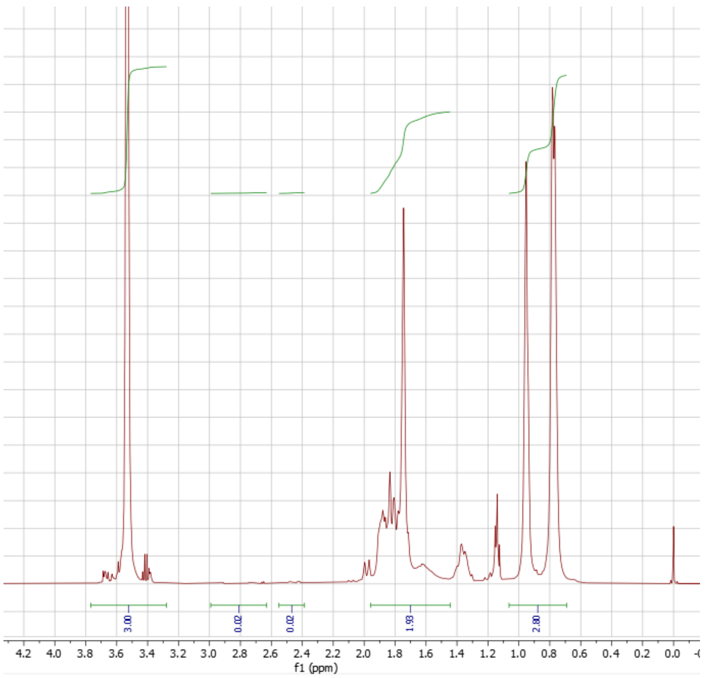

Figure S18. **^{1}H NMR of RAFT 15 kDa rPMMA.** ^{1}H NMR of RAFT 15 kDa rPMMA post-precipitation (d_6-acetone, 500 MHz)

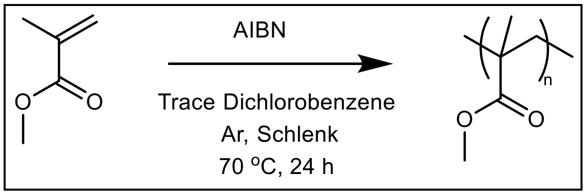

Figure S19: Reaction scheme for uncontrolled rMMA polymerization.

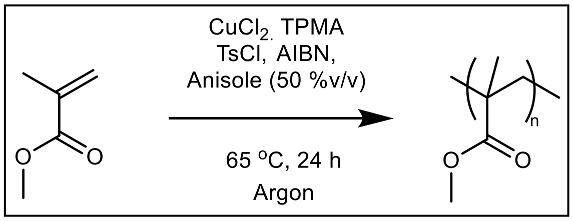

Figure S20: Reaction scheme for ATRP polymerization of MMA

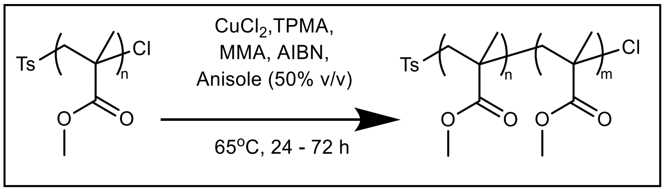

Figure S21: Reaction scheme for ATRP chain extension of ATRP macroinitiator with MMA.

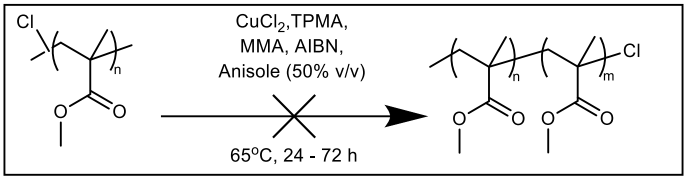

Figure S22: Reaction scheme for attempted ATRP chain extension of depolymerized PMMA, with MMA.

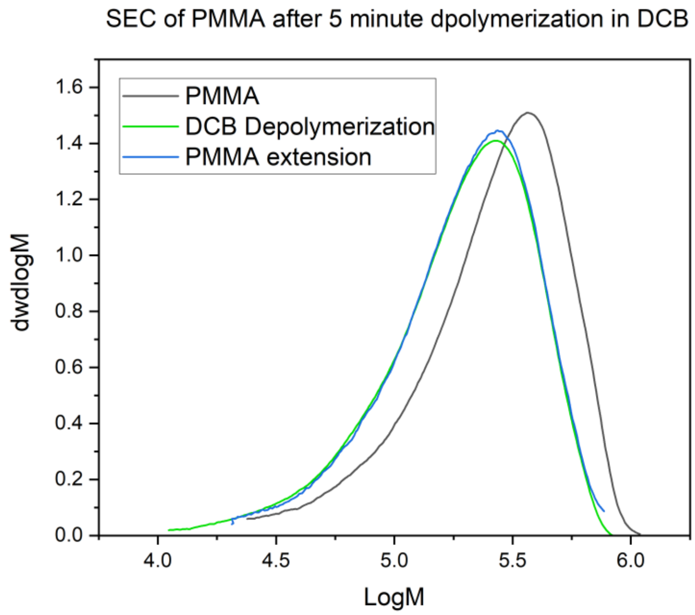

Figure S23: SEC for the failed ATRP chain extension of Cl-PMMA polymer. SEC (THF) of PMMA before and after 5 minutes of UV irradiation in DCB (green), showing decrease in LogM, and subsequent failed attempt at ATRP chain extension (blue).

Figures of SEC analyses of PMMA polymers

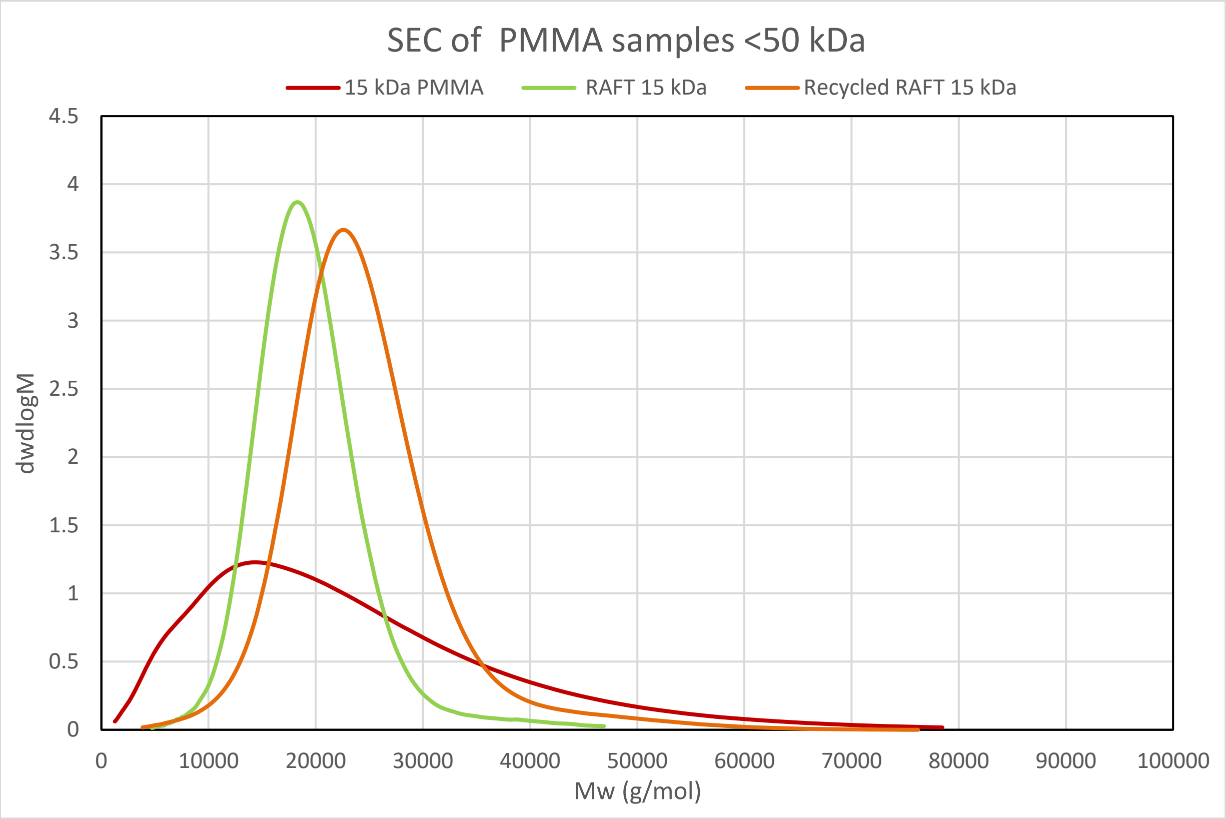

Figure S24: SEC analyses of PMMA polymers <50 kDa. SEC (THF) traces for polymers in this study with an M_n below 50 kDa (red - 15 kDa PMMA, green – RAFT 15 kDa PMMA, orange – recycled RAFT PMMA).

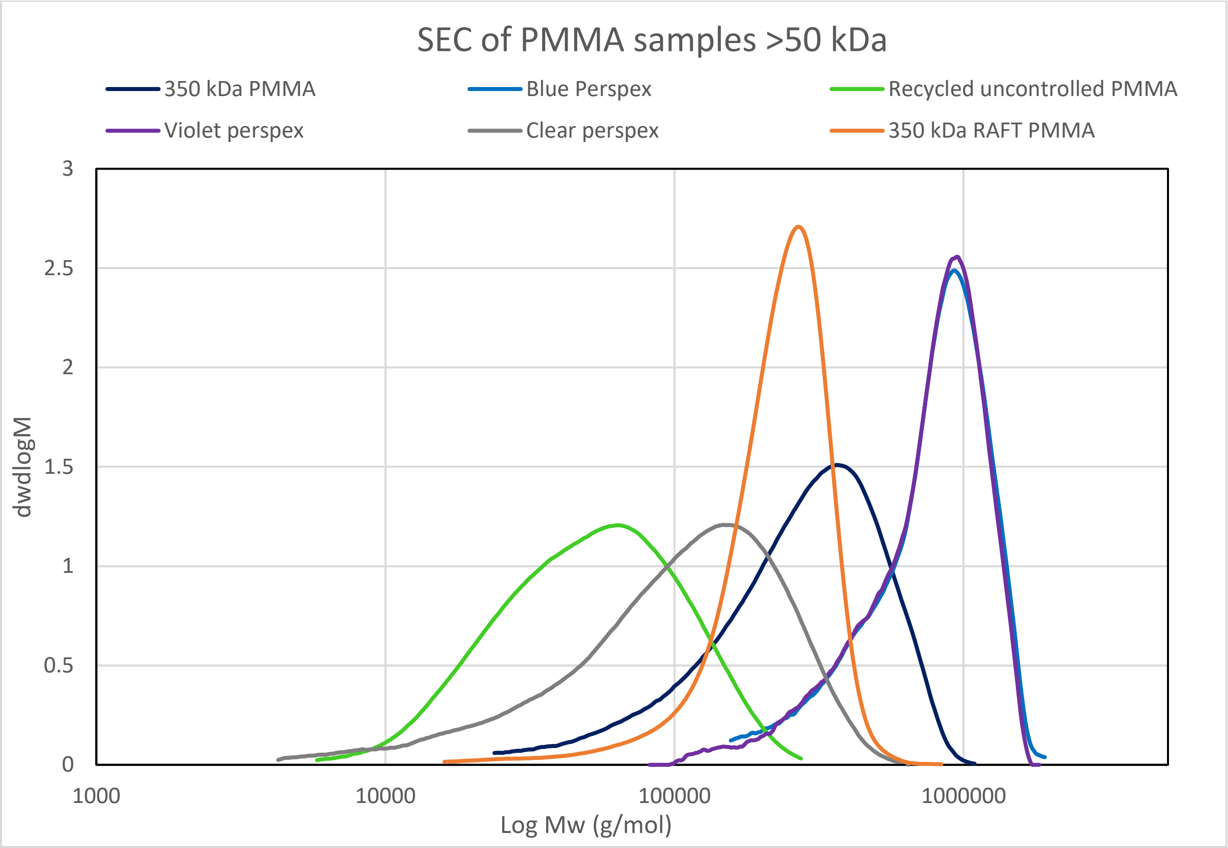

Figure S25: SEC analyses of PMMA polymers >50 kDa. SEC (THF) traces for polymers in this study with an M_n above 50 kDa (black – 350 kDa, blue – blue Perspex®, purple – violet Perspex®, grey – grey Perspex®, orange – 350 kDa RAFT PMMA, green – recycled uncontrolled PMMA).

Wavelength versus conversion

Table S1: Conversions (%) to monomer for different wavelengths of irradiation in DCB and benzonitrile at 50 mM, 140°C and 5 h.

Conversion (%)	365 nm	405 nm
DCB	49	48
Benzonitrile	36	29

Measurement of Critical Temperature (T_c) in different solvents

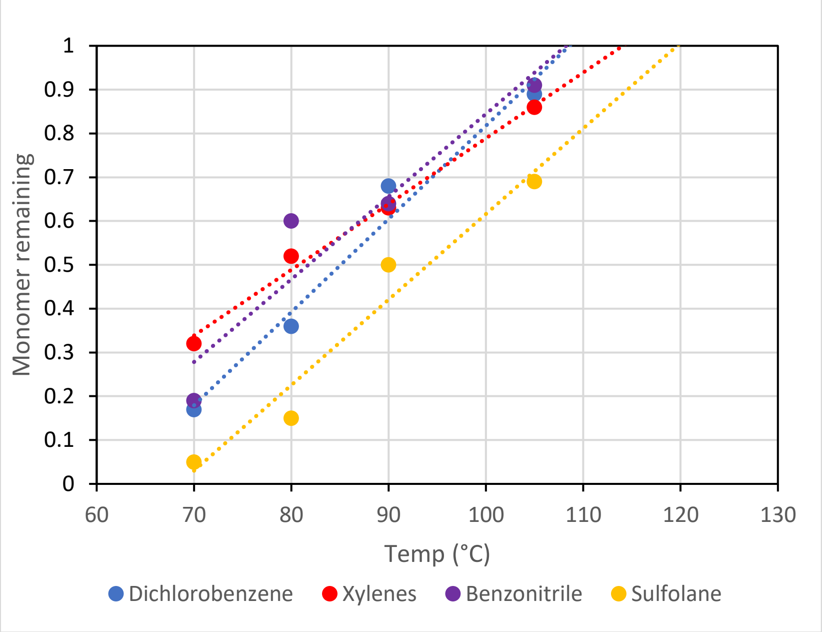

Figure S26. Plot of temperature versus $[M]_{eq}$ for PMMA polymerization in various solvents. Measured monomer remaining by ^1H NMR at equilibrium conversion at different temperatures (blue – dichlorobenzene, red – xylenes, purple – benzonitrile, yellow – sulfolane). Initial monomer concentration: $[M]_0$ = 0.2 M . Catalyst (AIBN) concentration: 1 mol %. Reaction time > 24 hours.

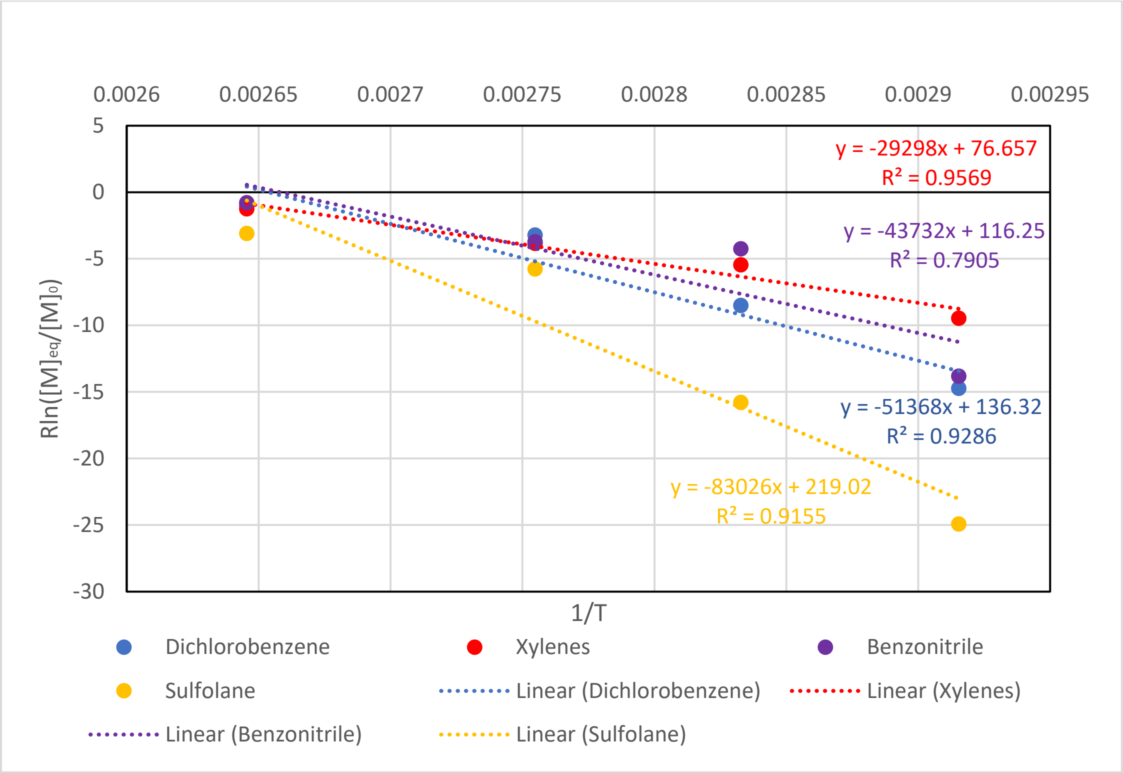

Figure S27. Plot of Theoretical equilibrium behavior Ln($[M]_{eq}/[M]_0$) as a function of temperature in selected solvents. Using $[M]_0$ and measured $[M]_{eq}$, Ln($[M]_{eq}/[M]_0$) was plotted against 1/T (blue – dichlorobenzene, red – xylenes, purple – benzonitrile, yellow – sulfolane) to allow calculation of dH, dS and therefore T_C by equation (1) and (2).

Solvent	dH	dS	T_C (K)	T_C (°C)	R^2
**Dichlorobenzene**	-51368	-136	377.7059	104.7059	0.93
**Xylenes**	-29298	-76.7	381.9817	108.9817	0.96
**Benzonitrile**	-43732	-116	377	104	0.79
**Sulfolane**	-83026	-219	379.1142	106.1142	0.92

Table S2. Thermodynamic parameters derived from Ln($[M]_{eq}/[M]_0$) as a function of temperature plot, including T_c, using equations (1) and (2).

Equation (1) : $Rln(\frac{[M]eq}{[M]_0} = \frac{1}{T} * \Delta H_p - \Delta S_p)$ Equation (2) : $T_C = \frac{\Delta H_p}{\Delta S_p}$

Proposed mechanism

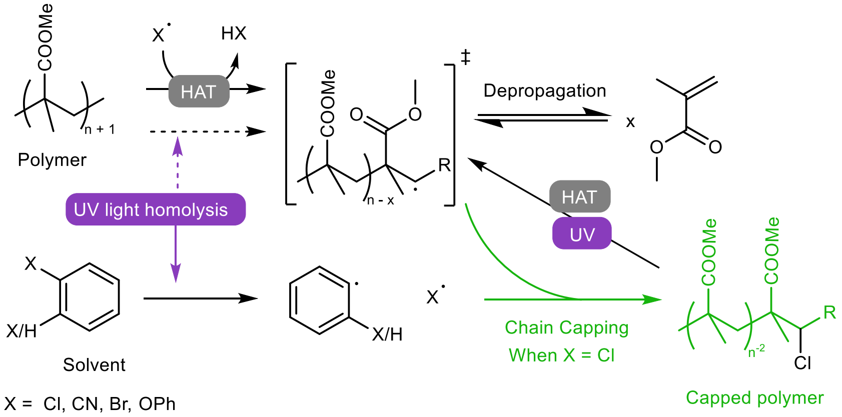

Figure S28. Scheme for the early-stage mechanistic theory for observations of the depolymerization. UV light causes homolysis and radical formation on the aromatic solvent. The dashed line represents PMMA homolysis which has not been ruled out. The solvent homolysis process produces a species capable of hydrogen atom transfer from the polymer backbone. The radical polymer species then undergoes either termination through reaction when high concentrations of solvent radicals are present (e.g. by Cl in DCB) or uncontrolled depolymerization releasing monomer (e.g. in benzonitrile). The Cl-capped polymer can then undergo re-initiation with UV or HAT.

UV-Vis spectrometry of PMMA polymers

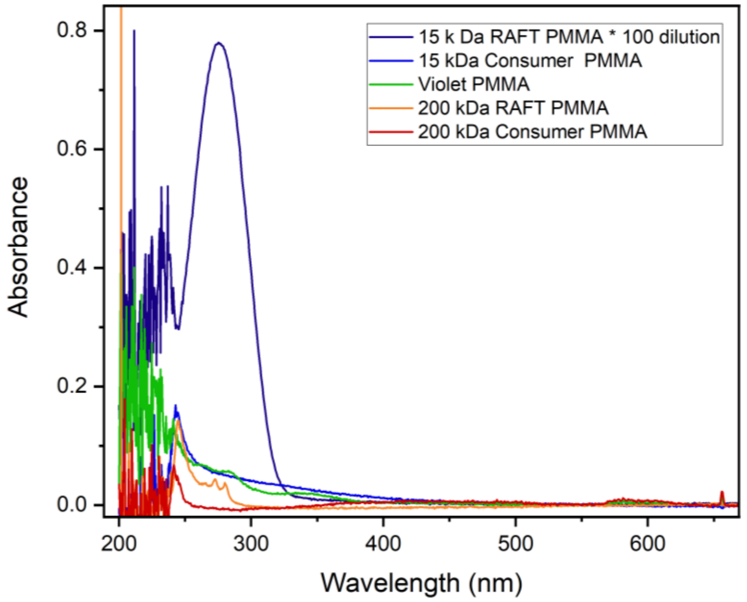

Figure S29: **UV-Vis spectrometry of PMMA polymers.** UV-Vis spectrometry of PMMA polymers in chloroform at 5mg/ml (excluding 15 kDa RAFT PMMA). No CTA absorbance is observed for the 15 kDa or 200 kDa consumer PMMA. The absorbance in violet Perspex® is attributed to dyes and additives in the plastic (red – 200 kDa PMMA, orange – 200 kDa RAFT PMMA, green – violet Perspex®, blue – 15 kDa PMMA, and navy – 15 kDa RAFT PMMA at 0.05 mg/mL).

Tables of conversions measured by ^{1}H NMR spectroscopy (Method 1)

Table S3: Investigating temperature and concentration relationships

Solvent	DCB	DCB	DCB	DCB	DCB	DCB	DCB	DCB	DCB	DCB	DCB	DCB	DCB	DCB	DCB	DCB	DCB	DCB	DCB	DCB	DCB
Monomer mg/mL	1.01	1.01	1.717	1.717	2.525	2.525	2.525	2.525	2.525	5.05	10.1	10.1	15.15	15.15	30.3	40.4	40.4	101	101	202	202
PMMA	300 kDa	15 kDa	15 kDa	15 kDa	300 kDa	15 kDa	15 kDa	15 kDa	300 kDa	15 kDa	15 kDa	15 kDa	15 kDa	300 kDa	15 kDa	15 kDa	300 kDa	300 kDa	15 kDa	15 kDa	300 kDa
Molarity (mM)	10	10	17	17	25	25	25	25	25	50	100	100	150	150	300	400	400	1000	1000	2000	2000
Temp	175	175	140	150	140	175	175	175	175	175	175	175	175	175	175	175	175	175	175	175	175
Time (min)	300	300	300	300	300	300	300	300	300	300	300	300	300	300	300	300	300	300	300	300	300
∫ $Monomer_{Vinyl}$	1	1	1	0.26	1	1	1	1	1	1	1	1	1	1	1	1	1	1	1	1	1
∫ $Polymer_{OMe}$ + $Monomer_{OMe}$	4.22	7.2	17.8	2.64	5.79	5.6	6.4	5.3	4.19	5.7	10.8	7.3	9.5	4.68	13.3	13.8	5.05	5.09	17.4	28.8	16.3
% Monomer	71.1	41.7	16.9	29.5	51.8	53.6	46.9	56.6	71.6	52.6	27.8	41.1	31.6	64.1	22.6	21.7	59.4	58.9	17.2	10.4	18.4

Table S4: Investigating Solvent -pt 1

Solvent	Cyrene	Sulfolane	Xylenes	Cyrene	Sulfolane	DCB	Xylenes	Diphenyether	Diphenyether	Mesitlyene	Ditertbuyl-phenol	Trichloro-benzene	Trichloro-benzene	Benzonitrile	Benzonitrile
PMMA	15 kDa	15 kDa	15 kDa	15 kDa	15 kDa	15 kDa	15 kDa	15 kDa	15 kDa	15 kDa	15 kDa	15 kDa	15 kDa	15 kDa	15 kDa
Molarity (mM)	25	25	25	25	25	25	25	35	35	25	25	25	25	25	25
Temp	175	175	140	175	175	175	140	175	140	175	175	140	175	140	175
Time (min)	300	300	300	300	300	300	300	300	300	300	300	300	300	300	300
∫ $Monomer_{Vinyl}$	1	1	1	1	1	1	1	1	1	1	1	1	1	1	1
∫ $Polymer_{OMe}$ + $Monomer_{OMe}$	45.6	Neg.	32.1	45.6	Neg.	5.3	32.1	6.4	47	Neg.	56	19.1	7.6	63	10.7
% Monomer	6.6	Neg.	9.3	6.6	Neg.	56.6	9.3	46.9	6.4	Neg.	1.8	15.7	39.5	4.8	28.0

Table S4: Investigating Solvent -pt 2

Solvent	NMP	Diphenylether	Dibromo-benzene	Sulfolane	NMP	Dibromo-benzene	NMP	Sulfolane	Methyl levullinate	p-Cymene	Vanilylalcohol	10% Sulfolane in DCB	50% Sulfolane in DCB
PMMA	15 kDa	15 kDa	15 kDa	15 kDa	15 kDa	15 kDa	15 kDa	15 kDa	15 kDa	15 kDa	15 kDa	15 kDa	15 kDa
Molarity (mM)	25	1000	25	25	25	25	25	25	25	25	25	25	25
Temp	175	175	175	175	175	140	140	140	140	160	160	140	140
Time (min)	300	1080	300	300		300	300	300	300	300	300	300	300
∫ $Monomer_{Vinyl}$	1	1	1	1	1	Neg	Neg	Neg	Neg	1	Neg	1	1
∫ $Polymer_{OMe}$ + $Monomer_{OMe}$	47	33	9.4	69	291	Neg	Neg	Neg	Neg	46	Neg	24.1	64.4
% Monomer	6.4	9.1	31.9	4.3	1.0	Neg	Neg	Neg	Neg	6.5	Neg	12.4	4.7

Investigating PMMA type

Solvent	DCB	DCB	DCB	DCB	DCB	DCB	DCB	DCB	DCB	DCB	DCB	DCB	DCB	DCB
Additional Variable	None	No UV	No UV	None	No UV	None	None	None	None	None	None	None	None	None
Monomer mg/mL	2.525	2.525	2.525	202	2.525	202	2.525	2.525	2.525	101	101	101	50.5	50.5
PMMA	300 kDa	RAFT PMMA 15 kDa	300 kDa	15 kDa	15 kDa	300 kDa	Clear Perspex	Blue Perspex	Violet Perspex	Violet Perspex	Blue Perspex	Clear Perspex	Violet Perspex	Violet Perspex
Molarity (mM)	25	25	25	2000	25	2000	25	25	25	1000	1000	1000	500	500
Temp	175	175	175	175	175	175	175	175	175	175	175	175	175	175
Time (min)	300	300	300	300	300	300	300	300	300	300	300	300	1080	1080
∫ $Monomer_{Vinyl}$	1	1	1	1	1	1	1	1	1	1	1	1	1	1
∫ $Polymer_{OMe}$ + $Monomer_{OMe}$	4.19	4.89	Neg	28.8	Neg	16.3	5.65	7.26	4.4	4.38	13.55	6.57	4.65	5.44
% Monomer	71.6	61.4	Neg	10.4	Neg	18.4	53.1	41.3	68.2	68.5	22.1	45.7	64.5	55.2
